# Prevalence and risk factors for brain white matter changes in young and middle-aged participants with Brain Dock (brain screening): a registry database study and literature review

**DOI:** 10.18632/aging.202933

**Published:** 2021-04-05

**Authors:** Tomohiro Yamasaki, Fusao Ikawa, Toshikazu Hidaka, Masashi Kuwabara, Shingo Matsuda, Iori Ozono, Masaaki Chiku, Naoyuki Kitamura, Tomoaki Hamano, Masahiro Akishita, Shuhei Yamaguchi, Hidekazu Tomimoto, Michiyasu Suzuki

**Affiliations:** 1 Postgraduate Clinical Training Center, Shimane University Hospital, Shimane, Japan; 2 Department of Neurosurgery, Shimane Prefectural Central Hospital, Shimane, Japan; 3 Department of Neurosurgery, Graduate School of Biomedical and Health Sciences, Hiroshima University, Hiroshima, Japan; 4 Department of Cardiovascular Medicine, Medical Check Studio Tokyo Ginza Clinic, Tokyo, Japan; 5 Department of Diagnostic Radiology, Kasumi Clinic, Hiroshima, Japan; 6 SmartScan, Inc., Tokyo, Japan; 7 Department of Geriatric Medicine, Graduate School of Medicine, University of Tokyo, Tokyo, Japan; 8 Hospital Bureau of Shimane Prefecture, Shimane, Japan; 9 Department of Neurology, Mie University Graduate School of Medicine, Mie, Japan; 10 Department of Advanced ThermoNeuroBiology, Yamaguchi University School of Medicine, Yamaguchi, Japan

**Keywords:** Brain Dock, cloud server, perivascular space, white matter change

## Abstract

This study aimed to determine the prevalence and risk factors for brain white matter changes in normal young and middle-aged participants who underwent Brain Dock (brain screening). We analyzed 5,000 consecutive healthy participants from the Brain Dock registry between August to December 2018. Age, sex, body mass index (BMI), medical history, deep subcortical white matter high intensity (DSWMH), periventricular high intensity (PVH), and enlargement of perivascular space (EPVS) were investigated in relation to age. The prevalence of DSWMH, PVH, and EPVS were 35.3%, 14.0%, and 17.8%, respectively. Multivariate logistic regression analyses for brain white matter changes were conducted. The significant risk factors in participants aged < 50 years were: age (OR:1.09, 95% CI:1.07-1.12), the female sex (1.29, 1.03-1.60), BMI obesity (1.86, 1.12-3.08), and hypertension (1.67, 1.18-2.35) for DSWMH; age (1.08, 1.04-1.13) and the female sex (1.56, 1.03-2.36) for PVH; and age (1.07, 1.05–1.10) and the female sex (0.77, 0.60-1.00) for EPVS. In conclusion, age was consistently identified as a significant risk factor in young and middle-aged participants. Some risk factors for brain white matter changes were identified even in young and middle-aged participants in this study. Further longitudinal studies should be done in the future.

## INTRODUCTION

White matter hyperintensities (WMH) are considered indicators of increased risk for dementia and stroke [1–7], depending on the degree of small vessel disease (SVD) [2, 6, 8]. Further, it is important to fully understand WMH data in young people before the onset of symptoms. However, to date, WMH reports in those < 50 years of age have been insufficient [9–11]. The perivascular space (PVS) has been reported to be an extension of the subarachnoid space surrounding the penetrating arteries [12]. Recently, the enlargement of the perivascular space (EPVS) was linked to lower cognitive performance in a healthy elderly population and in individuals with cerebral SVD [13]. This association may be related to the relationship between EPVS and the glymphatic/intramural periarterial drainage system reported by a large population-based setting [14]. However, there are many ambiguities regarding the significance of EPVS.

Having gained support by several organizations and municipalities since its inception in 1992, Brain Dock continues to be a widely available medical brain screening system in Japan [9]. Recently, Brain Dock has enabled remote diagnosis with automatically created databases using cloud storage; hence, online medical treatment in Japan has become increasingly promoted [15].

This study aimed to determine the prevalence of WMH and EPVS, and their corresponding risk factors in participants aged < 50 years by comparing their data from the Brain Dock registry with those of participants aged 50-59 years and ≥ 60 years.

## RESULTS

Table 1 shows the baseline characteristics, total prevalence rate, and prevalence in each age group. The average age was 49.1 ± 11.2 years and age range were from 13 to 91 years old. The median age was 49 years and interquartile ranges were 42 and 57 years old. The prevalence of obesity based on BMI, hypertension, and dyslipidemia were 24.4%, 16.3%, and 16.1%, respectively. The prevalence of grade I or higher WMH increased with advancing age, as did the proportion of participants with hypertension and dyslipidemia.

**Table 1:** Baseline characteristics of participants by age.

**Factor**	**Total**	**< 50 years**	**50-59 years**	**≥ 60 years**	***P* value**
	**n=5,000**	**n=2,668**	**n=1,408**	**n=924**	
Age, mean (SD), y	49.1 (11.2)	40.8 (6.4)	53.9 (2.9)	65.9 (5.1)	
Age, median (IQR), y	49 (42,57)	42 (37,46)	53 (51,56)	65 (62,69)	
Age, range, y	13-91	13-49	50-59	60-91	
Sex, female	2,296 (45.9%)	1,162 (43.6%)	695 (49.4%)	439 (47.5%)	0.001*
BMI (kg/m^2^)					0.005*
Skinny (< 18.5)	323 (6.5%)	189 (7.1%)	91 (6.5%)	43 (4.7%)	
Normal (18.5–25)	3,460 (69.2%)	1,874 (70.2%)	943 (67.0%)	643 (69.6%)	
Obesity1 (25–30)	1,029 (20.6%)	508 (19.0%)	310 (22.0%)	211 (22.8%)	
Obesity2 (> 30)	188 (3.8%)	97 (3.6%)	64 (4.5%)	27 (2.9%)	
Medical history					
Hypertension	814 (16.3%)	197 (7.4%)	281 (20.0%)	336 (36.4%)	< 0.001*
Diabetes mellitus	205 (4.1%)	36 (1.3%)	63 (4.5%)	106 (11.5%)	< 0.001*
Dyslipidemia	807 (16.1%)	267 (10.0%)	292 (20.7%)	248 (26.8%)	< 0.001*
Stroke	13 (0.3%)	7 (0.3%)	3 (0.2%)	3 (0.3%)	0.493
Surgery	1,647 (32.9%)	713 (26.7%)	543 (38.6%)	391 (42.3%)	< 0.001*
DSWMH					< 0.001*
Grade 0	3,237 (64.7%)	2,202 (82.5%)	787 (55.9%)	248 (26.8%)	
Grade I	1,382 (27.6%)	430 (16.1%)	520 (36.9%)	432 (46.8%)	
Grade II	359 (7.2%)	35 (1.3%)	97 (6.9%)	227 (24.6%)	
Grade III	22 (0.4%)	1 (0.0%)	4 (0.3%)	17 (1.8%)	
Grade IV	0 (0.0%)	0 (0.0%)	0 (0.0%)	0 (0.0%)	
PVH					< 0.001*
Grade 0	4,298 (86.0%)	2,562 (96.0%)	1,210 (85.9%)	526 (56.9%)	
Grade I	608 (12.2%)	102 (3.8%)	184 (13.1%)	322 (34.8%)	
Grade II	93 (1.9%)	4 (0.1%)	14 (1.0%)	75 (8.1%)	
Grade III	1 (0.0%)	0 (0.0%)	0 (0.0%)	1 (0.1%)	
Grade IV	0 (0.0%)	0 (0.0%)	0 (0.0%)	0 (0.0%)	
EPVS					< 0.001*
Grade 0	4,111 (82.2%)	2,347 (88.0%)	1,114 (79.1%)	650 (70.3%)	
Grade I	760 (15.2%)	305 (11.4%)	247 (17.6%)	208 (22.5%)	
Grade II	117 (2.3%)	15 (0.6%)	44 (3.1%)	58 (6.3%)	
Grade III	12 (0.2%)	1 (0.0%)	3 (0.2%)	8 (0.9%)	

BMI, body mass index; DSWMH, deep subcortical white matter high intensity; EPVS, enlargement of perivascular space; PVH, periventricular hyperintensity.

**Figure 1:**
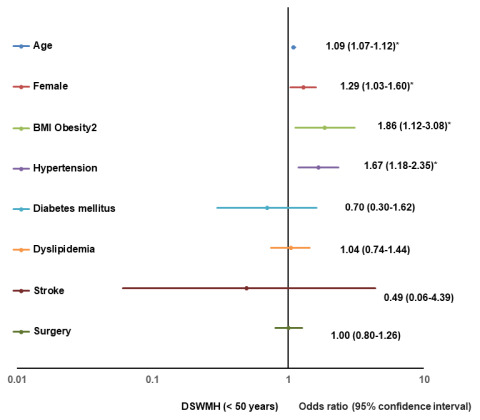
**Forest plot of multivariate logistic regression analysis for a positive change of DSWMH (< 50).** BMI, body mass index; DSWMH, deep subcortical white matter high intensity.

Tables 2–4 show the results of the multivariate logistic regression analysis for a positive change of grade I or higher in deep subcortical white matter high intensity (DSWMH), periventricular high intensity (PVH), and EPVS for participants aged < 50 years, 50-59 years, and ≥ 60 years, respectively. Significant risk factors for a positive change in DSWMH in participants aged < 50 years were age (1.09, 1.07–1.12), the female sex (1.29, 1.03–1.60), BMI > 30 kg/m^2^ (1.86, 1.12-3.08), and hypertension (1.67, 1.18–2.35); for participants aged 50-59 years, they were age (1.08, 1.04-1.12), hypertension (1.90, 1.43-2.54) and diabetes mellitus (3.16, 1.75-5.69); and for participants aged ≥ 60 years, however, analysis revealed only age as a risk factor (1.09, 1.06–1.13) (Table 2). Figure 1 shows the results of the multivariate logistic regression analysis for a positive change of grade I or higher in DSWMH for participants aged < 50 years, depicted as forest plots.

**Table 2:** Multivariate logistic regression analysis for the positive change of grade I or higher DSWMH in participants aged < 50 years, 50-59 years and ≥ 60 years.

	**The participants < 50 years**		**The participants 50-59 years**		**The participants ≥ 60 years**	
**Positive number (%)**	**466 (17.5%)**		**621 (44.1%)**		**676 (73.2%)**	
**factor**	**Odds ratios (95% CI)**	***P* value**	**Odds ratios (95% CI)**	***P* value**	**Odds ratios (95% CI)**	***P* value**
Age	1.09 (1.07-1.12)	< 0.001*	1.08 (1.04-1.12)	< 0.001*	1.09 (1.06-1.13)	< 0.001*
Sex, female	1.29 (1.03-1.60)	0.024*	1.14 (0.91-1.44)	0.251	1.24 (0.91-1.70)	0.175
BMI (kg/m^2^)						
Skinny (< 18.5)	0.88 (0.57-1.37)	0.571	0.98 (0.62-1.53)	0.920	1.27 (0.58-2.78)	0.551
Normal (18.5–25)	Reference		Reference		Reference	
Obesity1 (25–30)	1.16 (0.89-1.51)	0.275	0.85 (0.64-1.12)	0.248	0.96 (0.66-1.39)	0.833
Obesity2 (> 30)	1.86 (1.12-3.08)	0.016*	0.72 (0.41-1.26)	0.251	0.82 (0.34-1.98)	0.655
Medical history						
Hypertension	1.67 (1.18-2.35)	0.004*	1.90 (1.43-2.54)	< 0.001*	1.28 (0.91-1.80)	0.157
Diabetes mellitus	0.70 (0.30-1.62)	0.398	3.16 (1.75-5.69)	< 0.001*	0.99 (0.60-1.62)	0.963
Dyslipidemia	1.04 (0.74-1.44)	0.833	0.79 (0.60-1.05)	0.105	0.91 (0.64-1.28)	0.575
Stroke	0.49 (0.06-4.39)	0.528	3.10 (0.26-36.47)	0.369	0.70 (0.06-8.31)	0.780
Surgery	1.00 (0.80-1.26)	0.968	0.88 (0.70-1.10)	0.249	1.20 (0.88-1.64)	0.241

BMI, body mass index; CI, confidence interval; DSWMH, deep subcortical white matter high intensity.

*: A significant factor included in the 95% confidence interval.

**Table 3:** Multivariate logistic regression analysis for the positive change of grade I or higher PVH in participants aged < 50 years, 50-59 years and ≥ 60 years.

	**The participants < 50 years**		**The participants 50-59 years**		**The participants ≥ 60 years**	
**Positive number (%)**	**106 (4.0%)**		**198 (14.1%)**		**398 (43.1%)**	
**factor**	**Odds ratios (95% CI)**	***P* value**	**Odds ratios (95% CI)**	***P* value**	**Odds ratios (95% CI)**	***P* value**
Age	1.08 (1.04-1.13)	< 0.001*	1.16 (1.1-1.23)	< 0.001*	1.12 (1.08-1.15)	< 0.001*
Sex, female	1.56 (1.03-2.36)	0.037*	1.16 (0.84-1.62)	0.367	1.19 (0.89-1.60)	0.235
BMI (kg/m^2^)						
Skinny (< 18.5)	0.91 (0.40-2.03)	0.812	0.68 (0.33-1.43)	0.311	1.18 (0.60-2.32)	0.631
Normal (18.5–25)	Reference		Reference		Reference	
Obesity1 (25–30)	0.86 (0.50-1.49)	0.589	0.81 (0.54-1.22)	0.314	1.02 (0.73-1.45)	0.888
Obesity2 (> 30)	1.60 (0.64-4.03)	0.317	0.9 (0.43-1.85)	0.766	0.58 (0.24-1.37)	0.211
Medical history						
Hypertension	1.64 (0.87-3.09)	0.124	2.11 (1.46-3.05)	< 0.001*	2.23 (1.64-3.04)	< 0.001*
Diabetes mellitus	0.42 (0.05-3.32)	0.407	1.82 (0.96-3.43)	0.065	1.05 (0.67-1.64)	0.840
Dyslipidemia	0.82 (0.41-1.63)	0.562	1.13 (0.78-1.64)	0.528	0.84 (0.61-1.16)	0.285
Stroke	2.72 (0.27-27.22)	0.396	N/A		3.46 (0.29-41.85)	0.328
Surgery	1.17 (0.77-1.80)	0.458	0.83 (0.6-1.15)	0.269	1.16 (0.87-1.54)	0.312

BMI, body mass index; CI, confidence interval; N/A, not applicable; PVH, periventricular intensity.

*: A significant factor included in the 95% confidence interval.

**Table 4:** Multivariate logistic regression analysis for the positive change of grade I or higher EPVS in participants aged < 50 years, 50-59 years and ≥ 60 years.

**Positive number (%)** **factor**	**The participants < 50 years**		**The participants 50-59 years**		**The participants ≥ 60 years**	
	**321 (12.0%)**		**294 (20.9%)**		**274 (29.7%)**	
	**Odds ratios (95% CI)**	***P* value**	**Odds ratios (95% CI)**	***P* value**	**Odds ratios (95% CI)**	***P* value**
Age	1.07 (1.05-1.10)	< 0.001*	1.03 (0.98-1.08)	0.209	1.01 (0.98-1.04)	0.658
Sex, female	0.77 (0.60-1.00)	0.048*	0.66 (0.50-0.87)	0.003*	0.78 (0.57-1.05)	0.100
BMI (kg/m^2^)						
Skinny (< 18.5)	1.09 (0.66-1.82)	0.735	1.26 (0.73-2.17)	0.408	0.85 (0.40-1.80)	0.677
Normal (18.5–25)	Reference		Reference		Reference	
Obesity1 (25–30)	1.26 (0.94-1.69)	0.123	0.97 (0.70-1.34)	0.849	0.82 (0.57-1.18)	0.294
Obesity2 (> 30)	1.53 (0.84-2.76)	0.163	0.83 (0.43-1.59)	0.567	1.42 (0.64-3.17)	0.392
Medical history						
Hypertension	1.22 (0.81-1.83)	0.344	1.26 (0.90-1.76)	0.179	1.71 (1.25-2.35)	0.001*
Diabetes mellitus	1.50 (0.67-3.39)	0.324	1.32 (0.73-2.40)	0.360	1.12 (0.71-1.75)	0.630
Dyslipidemia	0.88 (0.60-1.30)	0.525	1.04 (0.75-1.44)	0.832	0.96 (0.69-1.34)	0.806
Stroke	N/A		N/A		N/A	
Surgery	1.00 (0.76-1.30)	0.975	1.08 (0.82-1.41)	0.589	1.16 (0.86-1.55)	0.329

BMI, body mass index; CI, confidence interval; EPVS, enlargement of perivascular space; N/A, not applicable.

*: A significant factor included in the 95% confidence interval.

**Figure 2:**
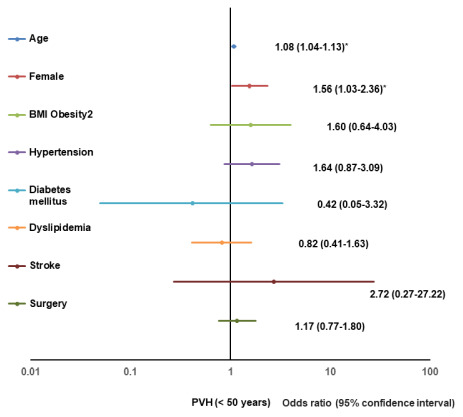
**Forest plot of multivariate logistic regression analysis for a positive change of PVH (< 50).** BMI, body mass index; PVH, perivascular high intensity.

Significant risk factors for a positive change of grade I or higher PVH in participants aged < 50 years were age (1.08, 1.04–1.13) and the female sex (1.56, 1.03–2.36); for participants aged 50-59 years, they were age (1.16, 1.1-1.23) and hypertension (2.11, 1.46-3.05); and for participants aged ≥ 60 years, they included age (1.12, 1.08–1.15) and hypertension (2.23, 1.64–3.04) (Table 3). Figure 2 shows the results of the multivariate logistic regression analysis for a positive change of grade I or higher in PVH for participants aged < 50 years, depicted as forest plots.

**Figure 3:**
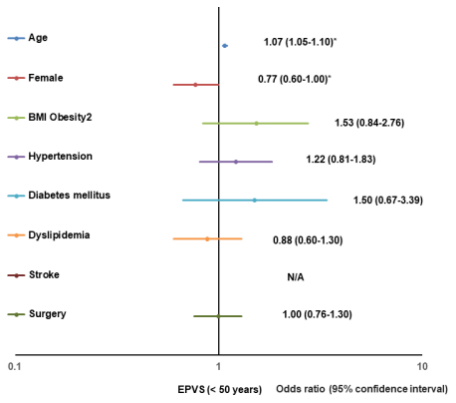
**Forest plot of multivariate logistic regression analysis for a positive change of EPVS (< 50).** BMI, body mass index; EPVS, enlargement of perivascular space; N/A, not applicable.

The significant risk factors for positive changes in EPVS in participants aged < 50 years were age (1.07, 1.05–1.10) and the female sex (0.77, 0.60-1.00); for participants aged 50-59 years, it was the female sex (0.66, 0.50-0.87); for participants aged ≥ 60 years, it was hypertension (1.71, 1.25–2.35) (Table 4). Figure 3 shows the results of the multivariate logistic regression analysis for a positive change of grade I or higher in EPVS for participants aged < 50 years, depicted as forest plots.

### Prevalence of WMH among young and middle-aged participants

The prevalence of grade I or higher DSWMH was 17.5% among participants aged < 50 years, in contrast to a prevalence of 73.2% among those aged ≥ 60 years. The prevalence of the same change in PVH was 4.0% among participants aged < 50 years, as compared to a prevalence of 43.1% among participants aged ≥ 60 years.

### Prevalence of WMH among young and middle-aged according to a literature review

Review of the literatures indicated the prevalence of WMH as DSWMH and PVH among healthy individuals of < 50 years of age ranged from 3.0% to 66.7% (Table 5) [11, 16–22].

**Table 5:** Summary of literature review of prevalence of white matter high intensity in normal young and middle-aged participants.

	**Reference**	**Year**	**Country**	**MRI**	**Number**	**Normal or disease**	**Age, year**		**Total**	**DSWMH**	**PVH**
				**Tesla**			**Mean (SD)**	**Range**	**Prevalence (%)**		
1	Masana et al. [11]	2003	Japan	1.5	711	Normal	N/A	17–50	21 (3.0%)	N/A	N/A
2	Hopkins, R. O. [16]	2006	U.S.	1.5	243	Normal	37.0 (13.5)	16–65	13 (5.3%)	N/A	N/A
3	Wen, W. [17]	2009	Australia	1.5	428	Normal	46.7 (1.4)	44-48	218 (50.4%)	146 (34.1%)	126 (29.4%)
4	McGuire, S. A. [18]	2014	U.S.	3	148	Normal	34.6 (5.8)	26-50	2.6 (3.1)^a^	N/A	N/A
5	Huang, C. C. et al. [19]	2018	China	3	102	Normal	26.5 (3.4)	21–35	0.11 (0.26)^b^	0.05 (0.13)^b^	0.06 (0.23)^b^
					89	Normal	49.6 (7.1)	36-59	0.27 (0.51)^b^	0.05 (0.08)^b^	0.21 (0.48)^b^
6	Williamson, W. et al. [20]	2018	United Kingdom	3	125	Normal	24.7 (5.0)	18–40	20.9 (7.9)^a^	N/A	N/A
7	Keřkovský, M. et al. [21]	2019	Germany	1.5	60	Normal	34.5 (8.3)	21–62	40 (66.7%)	N/A	N/A
8	Wadhwa, R. et al. [22]	2019	Netherlands	3	108	Normal	22.2 (3.9)	12–30	N/A	51 (47.2%)	19 (17.6%)
					131	People with family history of BD	20.3 (5.7)	12–30	N/A	59 (45.0%)	34 (26.0%)
					47	Patients with BD	25.4 (3.5)	12–30	N/A	20 (42.6%)	15 (31.9%)

BD, bipolar disorder; DSWMH, deep subcortical white matter high intensity; N/A, not applicable; PVH, periventricular hyperintensity; U.S., United States of America.

a: Lesion count, mean (SD), b: WMH volume, cc (SD).

### Prevalence of EPVS among young and middle-aged participants

The prevalence of EPVS among patients aged < 50 years was 12.0%, and that among participants aged ≥ 60 years was 29.7%.

### Prevalence of EPVS among young and middle-aged according to a literature review

Table 6 presents the prevalence rate of EPVS [23–30]. There are no reports on the prevalence of EPVS among young and middle-aged individuals aged < 50 years; hence, we only included data on participants without age restrictions. The evaluation was mainly based on lesion count: from 2.1 to 30.8 in a healthy population.

**Table 6:** Summary of literature review of prevalence of enlargement of perivascular space.

	**Reference**	**Year**	**Country**	**MRI**	**Number**	**Normal or disease**	**Age, year**		**EPVS**
			**(Study name)**	**Tesla**			**Mean (SD)**	**Range**	**Counts, mean (SD)**
1	Völzke H. et al. [23, 24]	2011	Germany (SHIP-2)	3	385	Normal	68.5 (6)	30–90	8.2 (7.3)
2	Seiler, S. et al. [24, 25]	2014	Austria (ASPS-Fam)	1.5	275	Normal	63.4 (10.9)	N/A	30.8 (2.4)
3	Ikram, MA. et al. [24, 26]	2017	Netherland (RS)	3	1,409	Normal	73.2 (7.5)	N/A	16.7 (9.5)
4	Hilal, S. et al. [24, 27]	2017	Singapore (EDIS)	1.5	529	Normal	70.4 (6.6)	≥ 40	8.9 (7.1)
5	Hilal, S. et al. [24, 28]	2017	Hong Kong (CU-RISK)	1.5	208	Normal	71.4 (5.3)	≥ 65	2.1 (2.8)
6	Cavallari, M. et al. [29]	2018	U.S.	1.5	30	Normal	48^a^	27–63	11.5 (3–26)^b^
					30	Multiple sclerosis	50^a^	27–68	11.0 (2–26)^b^
7	Niazi, M. et al. [30]	2018	U.S.	3	15	Normal	66.3 (9.5)	55–89	N/A
					14	MCI	71.9 (6.2)		N/A

ASPS-Fam: Austrian Stroke Prevention Family Study, CU-RISK: Chinese University of Hong Kong–Risk Index for Subclinical Brain Lesions in Hong Kong, EDIS: Epidemiology of Dementia in Singapore, EPVS: enlargement of perivascular space, MCI: mild cognitive impairment, N/A: not applicable, RS: Rotterdam Study, SHIP: Study of Health in Pomerania, US: United States of America.

a: Age, median, year, b: Counts, median (range), c: Rate of EPVS volume fraction, mean (SD).

## DISCUSSION

### Prevalence and risk factors of WMH in young and middle-aged people

The present study found that the prevalence of grade I or higher DSWMH and PVH were 17.5% and 4.0%, respectively, among participants aged < 50 years. Although the prevalence of WMH among young and middle-aged individuals ranged widely, these results fell within previously reported ranges [11, 16–22]. Because two studies used automated tools [17, 20], higher prevalences of WMH lesions were shown in these two studies. Using a 3D fluid-attenuated inversion recovery (FLAIR) sequence [21], another investigation reported a relatively higher prevalence of WMH lesions than did studies that used other sequences, furthermore a study on individuals with bipolar disorders showed a higher prevalence of DSWMH lesions than did the other reports [22]. The prevalence of DSWMH and PVH in patients with bipolar disorder who were < 30 years of age was > 40% and as high as 25%, respectively [22]. Excluding these reports, the prevalence rate of WMH in young and middle-aged adults ranged from 3.0% to 5.3%.

The present study found age to be a common risk factor for DSWMH and PVH regardless of the age group or the location of WMH. Female sex was a common risk factor for DSWMH and PVH among participants aged < 50 years, regardless of the location of WMH. Hypertension was a risk factor for DSWMH among adults aged < 50 years and PVH in those of ≥ 60 years of age. Severe obesity was a risk factor for DSWMH among participants aged < 50 years. Age and hypertension are well-known risk factors for DSWMH, as well as PVH [11]. Obesity increases the number of WMH lesions [20] because an increase in BMI decreases myelin and iron content, and increases water content [31]. Although no significant differences between men and women were found by earlier studies on WMH [32] and PVH [11] prevalence, our present study found the female sex to be a significant risk factor for the prevalence of both DSWMH and PVH. This result was consistent with previous studies of WMH [33, 34]. Furthermore, a meta-analysis found the prevalence of Alzheimer’s disease among females to be 1.9 times that of males [35], suggesting an association between WMH changes among young and middle-aged women and their future development of Alzheimer’s disease or cognitive decline. A longitudinal study is required to confirm this hypothesis.

WMH is associated with vascular risk factors and is often considered indicative of SVD. WMH accelerates during brain aging throughout adulthood in the general population as a result of vascular risk factors; this can be prevented or delayed by controlling for the vascular risk factors [36]. Study of a healthy Chinese Han population found a particularly high rate of increases in WMH, especially between the ages of 50-60 years [19]. Therefore, the presently identified factors related to WMH increases in individuals aged < 50 years could be relevant to determining the extent of WMH changes during the later period. An autopsy study found that DSWMH was more frequently associated with cerebral ischemia than with PVH [37]. Alternatively, a study of patients with transient ischemic attack or minor stroke found that increased WMH, especially PVH lesions, was associated with cognitive decline [38]. Smoking has been reported to be another risk factor for WMH, especially for PVH in younger age groups, and has been reported to exacerbate WMH [39–41]. Inflammation is also another risk factor for WMH [42]; however, unfortunately we could not include these factors in our study. Our population should be monitored by a longitudinal study to examine the relationship between WMH and cerebral ischemia and cognitive decline.

### Prevalence and risk factors of EPVS in young and middle-aged people

We found the prevalence of EPVS among participants aged < 50, 50-59, and ≥ 60 years to be 12.0%, 21.0%, and 29.7% respectively. The average number of lesions ranged from 0.52 to 1.93 when the prevalence has calculated the average number of lesions according to our grading system. These rates were lower than those previously reported [23–30]. We attribute this discrepancy to our relatively young patient population.

Whether EPVS is clinically significant remains controversial and should, therefore, not be referred to as a lesion; however, some studies have associated more prominent PVS with worse cognitive function [43]. It appears likely that EPVS indicates the obstruction by protein and cell debris and consequently the stagnation of fluid drainage, further indicating the failure of the glymphatic and intramural periarterial drainage system. The densities of EPVS in a patient with mild cognitive impairment (MCI) was found to be significantly higher than that observed in normal participants, [30]. Additionally, lower water diffusivity along the PVS was associated with the severity of Alzheimer’s disease [44]. According to another report, EPVS of ≥ 3 mm was associated with cognitive decline [24]. Therefore, EPVS density may be a promising imaging biomarker for the diagnosis of MCI [30].

In the present study, age, the male sex, and hypertension were found to be risk factors for the prevalence of EPVS. The result concerning age agrees with the findings of earlier studies [45, 46]. In further agreement with our results, a previous report found that the severity of EPVS to be associated with age and hypertension [46], and a close relationship between EPVS and SVD has also been previously reported [47]. A combination of vascular dysfunction, inflammation, and blood brain barrier dysfunction likely underlie SVD and have devastating effects on brain health. However, the timing and contribution of these events to the pathophysiology of SVD remain unconfirmed. MRI has revealed imaging biomarkers for SVD, including EPVS, which correlate with the SVD burden. Nevertheless, how PVS becomes enlarged in patients with SVD and its role in the pathogenesis of the disease remains to be determined [47]. We speculate the following reasons for our identification of the male sex as a risk factor for EPVS among young and middle-aged participants: natural sleep improves interstitial solute clearance, and the glymphatic system is mainly active during sleep [48, 49]. That poor sleep quality is more common among men than women can be attributed to the observations that the male sex and obesity are risk factors for sleep-disordered breathing [50], as well as to the finding that young and middle-aged males have a higher incidence of arteriosclerosis and stroke than do women who have not undergone menopause [51–53].

### Limitations

We have several limitations in this study. First, all scans were obtained from 1.5T MRI equipment. Therefore, there is a suspicion that the comparison with the other 3T MRI equipment studies is not accurate. Second, medical history was determined based on a questionnaire by participants; therefore, it may not be completely accurate. Third, there is a lack of data for smoking and inflammation in this study. Fourth, this study is cross-sectional and the causality of the observed relationships cannot be inferred. Longitudinal follow-up will be required to determine the clinical significance of the observed findings.

## CONCLUSIONS

In the registry database using the remote Brain Dock, we found that age, the female sex, hypertension, and obesity were significant risk factors for brain white matter changes even in young and middle-aged participants. For EPVS, age and male were significant risk factors in young and middle-aged participants. Further big data analysis and long-term follow-up are necessary to confirm the pathological significance and changes in the EPVS.

## MATERIALS AND METHODS

### Ethical statement

The study was approved by the Clinical Research and Investigational Review Board of the Shimane Prefectural Central Hospital (NO. R19-040). Individual data were anonymized, routinely collected for Brain Dock, and posed no risk to the participants. Thus, the requirement for individual informed consent was waived, instead an opt-out method was used as informed consent for this study.

### Data source

The Japan Brain Dock Society was founded in 1992 for the prevention of stroke and detection of asymptomatic brain lesions. In this study, we focused on the detection of WMH in participants in Brain Dock.

**Figure 4:**
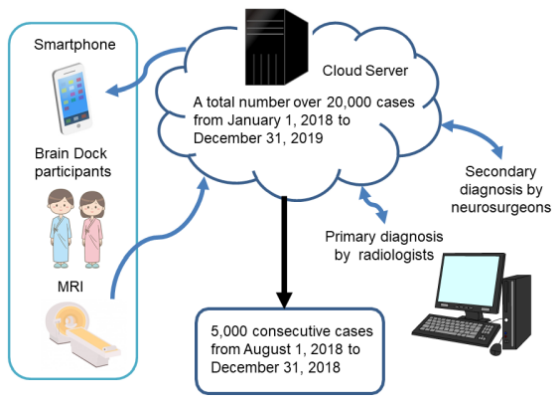
**Selection process of participants.**

We analyzed 5000 cases diagnosed by a remote image diagnosis system “LOOKREC”, that was developed by Medical Network Systems incorporated (MNES Inc.). In the remote image diagnosis system, first, an MRI examination of the subject’s brain was performed at the Medical Check Studio Tokyo Ginza Clinic and SmartScan, Inc., and the examined image was uploaded to the cloud. After a primary diagnosis by the radiologists in MNES Inc., neurosurgeons at the Hiroshima University, Kagoshima University, or Tokushima University carried out the diagnosis and uploaded the secondary diagnostic results to the cloud. Based on these results, a final diagnosis was uploaded to the cloud from the Medical Check Studio Tokyo Ginza. Thereafter, participants could confirm their own final diagnosis and check their data that were accumulated on the cloud server; therefore, the database of participants was structured simultaneously (Figure 4). The image server is built on cloud (Google Cloud Platform), does not include personal information, and ensures security through virtual private network (VPN) communication using secure sockets layer (SSL)/transport layer security (TLS) and two-step authentication.

### Neuroimaging

All scans were obtained from 1.5 T MRI equipment (ELAN, Canon Medical, Japan). The neuroimaging protocol included the following sequences: T1-weighted, T2-weighted, T2* weighted, FLAIR sequences, diffusion-weighted image.

### Patient and data

We analyzed 5000 cases from August 21 to December 28, 2018 in the Brain Dock database. Age, sex, BMI, medical history (hypertension, diabetes mellitus, dyslipidemia, stroke, dementia, and history of surgeries), DSWMH, PVH, and EPVS were investigated. Patient BMI was categorized as follows: skinny (S), < 18.5 kg/m^2^; normal (N), 18.5 kg/m^2^ to under 25 kg/m^2^; obesity1 (O1), 25 kg/m^2^ to under 30 kg/m^2^; obesity2 (O2), 30 kg/m^2^ or more. The medical history (hypertension, diabetes mellitus, dyslipidemia, stroke, dementia, and history of surgeries) was determined based on a questionnaire completed by the participants. Medical history is judged as positive if the participant is being treated or pointed out for hypertension, hyperlipidemia, or diabetes mellitus by the medical checkup. WMH was defined as deep subcortical (DSWMH) or periventricular (PVH) hyperintensity lesion on FLAIR. DSWMH was classified as Grade 0–IV. Grade 0: absent; Grade I: punctate foci, diameter ≤ 3 mm, boundary clear; Grade II: diameter > 3 mm, punctate or discrete foci; Grade III: large confluent foci, boundary unclear; Grade IV: confluence widely distributed in most of the white matter. PVH was classified as Grade 0– IV. Grade 0: absent or ‘rims’ only; Grade I: localized lesions such as ‘caps’; Grade II: extended along the whole periventricular area; Grade III: irregular PVH extending into the deep white matter; Grade IV: extending throughout deep and subcortical white matter [54, 55]. EPVS were defined as ovoid or linear lesions visible as hypointense regions on T1-weighted and as hyperintense on T2-weighted images and were considered to be dilated if their size was ≥ 2 mm. EPVS was classified as grade 0–III based on the number of lesions. Grade 0: None; Grade I: 1–5; Grade II: 6–10; Grade III: ≥ 11 [56]. We defined a positive change of DSWMH, PVH, and EPVS as grade I or higher change in all lesions. Age was categorized into < 50 years, 50-59 years, and ≥ 60 years.

### Review of the literatures

The literature search on the prevalence of WMH among healthy young and middle-aged individuals included articles published between January 2000 and March 2020. The search was performed on PubMed with publications restricted to those written in the English language, and the last search date was March 31, 2020. Keywords and free text searches used combinations of the following keywords: white matter hyperintensity, healthy populations, young adult, middle age, and MRI. All reference sections of eligible studies and relevant reviews were further reviewed for potential studies. If a study generated multiple publications, the most current report was considered for the analysis.

### Statistical analysis

Categorical variables were compared using the chi-square test or Fisher’s exact test. Continuous variables were compared using the t-test or Mann-Whitney U test, as appropriate. *P*-values < 0.05 were considered to indicate statistical significance. Missing variables were treated as deficit data, not affecting other variables. Multivariate logistic regression analysis for the positive change, as grade I or higher in DSWMH, PVH, and EPVS, was performed on all participants and groups of participants aged < 50 years, 50-59 years, and ≥ 60 years. Odds ratio (OR) and 95% confidence interval (CI) were calculated for each group. For multivariable logistic regression analysis, independent variables were selected based on existing literature, and a no variable selection method, such as stepwise selection was applied. All statistical analyses were performed using JMP® Pro15 software (SAS Institute Inc., Cary, NC, USA).
